# A serum protein signature of *APOE* genotypes in centenarians

**DOI:** 10.1111/acel.13023

**Published:** 2019-08-05

**Authors:** Paola Sebastiani, Stefano Monti, Melody Morris, Anastasia Gurinovich, Tanaka Toshiko, Stacy L. Andersen, Benjamin Sweigart, Luigi Ferrucci, Lori L. Jennings, David J. Glass, Thomas T. Perls

**Affiliations:** ^1^ Department of Biostatistics Boston University School of Public Health Boston Massachusetts; ^2^ Bioinformatics Program Boston University Boston Massachusetts; ^3^ Division of Computational Biomedicine, Department of Medicine Boston University School of Medicine Boston Massachusetts; ^4^ Novartis Institutes for Biomedical Research Cambridge Massachusetts; ^5^ Translational Gerontology Branch National Institute on Aging Baltimore Maryland; ^6^ Geriatrics Section, Department of Medicine, School of Medicine and Boston Medical Center Boston University Boston MA

**Keywords:** APOE, centenarian, cognitive function, genotypes, protein, SomaLogic

## Abstract

The discovery of treatments to prevent or delay dementia and Alzheimer's disease is a priority. The gene *APOE* is associated with cognitive change and late‐onset Alzheimer's disease, and epidemiological studies have provided strong evidence that the *e*_2_ allele of *APOE* has a neuroprotective effect, it is associated with increased longevity and an extended healthy lifespan in centenarians. In this study, we correlated *APOE* genotype data of 222 participants of the New England Centenarian Study, including 75 centenarians, 82 centenarian offspring, and 65 controls, comprising 55 carriers of *APOE* *e*_2_, with aptamer‐based serum proteomics (SomaLogic technology) of 4,785 human proteins corresponding to 4,137 genes. We discovered a signature of 16 proteins that associated with different *APOE* genotypes and replicated the signature in three independent studies. We also show that the protein signature tracks with gene expression profiles in brains of late‐onset Alzheimer's disease versus healthy controls. Finally, we show that seven of these proteins correlate with cognitive function patterns in longitudinally collected data. This analysis in particular suggests that Baculoviral IAP repeat containing two (BIRC2) is a novel biomarker of neuroprotection that associates with the neuroprotective allele of *APOE*. Therefore, targeting *APOE* *e*_2_ molecularly may preserve cognitive function.

## 1 INTRODUCTION

The prevalence of late‐onset Alzheimer's disease (LOAD), dementia, and cognitive impairment is growing with the aging population, and reliable plasma/serum biomarkers that can be used for pre‐clinical diagnosis, monitoring and prediction of cognitive decline, onset of cognitive impairment, and for suggesting therapeutic targets are lacking (Cheng et al., 2018; Xia, Jiang, McDermott, & Han, 2018). Recent studies have shown that the development of drugs related to genetic findings is more likely to be successful than average (Nelson et al., 2015), and since the gene *APOE* is the most robust genetic variant associated with cognitive change and LOAD, it is an obvious target. The gene's three prevalent alleles, *e*_2_, *e*_3_ and *e*_4_, are defined by combinations of genotypes of the single nucleotide polymorphisms (SNPs) rs7412 and rs429358. The ancestral allele *e*_4_ is a well‐established risk factor for LOAD, cardiovascular events, and poor cognitive function (Corder et al., 1993; Liu, Liu, Kanekiyo, Xu, & Bu, 2013). The *e*_3_ allele is the “neutral allele” in many ethnicities, while *e*_2_ is the least common allele that emerged as a longevity variant when Schachter et al. noted an increased frequency of *e*_2_ in French centenarians (Schachter et al., 1994). Since then, several studies have provided evidence that *e*_2_ has a beneficial neuroprotective effect (Kim et al., 2017), decreases neuroinflammation (Dorey, Chang, Liu, Yang, & Zhang, 2014), and promotes longevity (Sebastiani, Bae, et al., 2017; Sebastiani, Gurinovich, et al., 2017; Sebastiani et al., 2018) and healthy aging (Kulminski et al., 2016; Wu & Zhao, 2016). Therefore, we hypothesize that targets of this allele may lead to the discovery of treatments that help maintain good cognitive function and escape cognitive impairment with aging.

Despite multiple research efforts, the biological mechanisms associated with variants of *APOE*, particularly the *e*_2_ allele, are still unclear. One strategy to understand the paths linking *APOE* alleles to phenotypes is to examine their biological products, starting, for example, from the list of genes that are in *cis* and in *trans* with *APOE* alleles. The challenge of these analyses is the tissue specificity of the results, the relative rarity of carriers of the *e*_2_ allele in the population, and the fact that relevant tissues like brain are not easily accessible. Recent studies have shown that the APOE protein and additional proteins associated with *APOE* genotypes can be detected in serum (Emilsson et al., 2018) and plasma (Muenchhoff et al., 2017; Rezeli et al., 2015; Simon et al., 2012; Sun et al., 2018), thus opening the way to new research avenues to both decipher the mechanisms linking genotypes to phenotypes, and to provide sensitive, noninvasive biomarkers of AD or progression of cognitive decline or protection from these phenotypes.

In this study, we leveraged the over‐representation of *e*_2_ in centenarians and their offspring to correlate *APOE* genotype data of 222 participants of the New England Centenarian Study, including 75 centenarians, 82 centenarian offspring, and 65 controls, comprising 55 carriers of *APOE* *e*_2_, with aptamer‐based serum proteomics (SomaLogic technology) of 4,785 human proteins corresponding to 4,137 genes. We discovered and replicated a list of 16 proteins that associate with different *APOE* genotypes and map to different gene expression profiles in brains of LOAD and healthy controls. We also showed that some of the proteins in the signature correlate with patterns of cognitive function.

## 2 MATERIALS AND METHODS

### 2.1 Study Populations

#### 2.1.1 NECS

The New England Centenarian Study (NECS) is a study of centenarians, their long‐lived siblings, offspring, and controls who are either individuals with one parent who died age 72–74 (average life expectancy for the centenarian birth cohort) or spouses of centenarian offspring (Sebastiani & Perls, 2012). The study began by recruiting centenarians in the Boston metropolitan area in 1994 and expanded in the late 1990s to include North America and English speaking countries. The age of participants is carefully validated (Young et al., 2010), and participants are followed up annually to assess their health, physical, and cognitive functions. The cognitive assessment in centenarians is administered annually using the 37‐point Blessed Information‐Memory‐Concentration (BIMC) test (Kawas, Karagiozis, Resau, Corrada, & Brookmeyer, 1995), and in centenarians' offspring and controls cognitive testing is performed every other year using the Telephone Interview for Cognitive Status (TICS). TICS is based on 12 items with a maximum of 51 points and assesses orientation to time and place, episodic memory, language, and working memory (Brandt, Spencer, & Folstein, 1988). An abbreviated version of TICS that includes the tasks of counting backwards, subtracting sevens, and immediate and delayed word list recall with a maximum of 27 points was validated against detailed in‐person neuropsychological testing and clinician adjudication (Crimmins, Kim, Langa, & Weir, 2011). All subjects provided informed consent approved by the Boston University Medical Campus IRB.

#### 2.1.2 InChianti

The Invecchiare in Chianti (InCHIANTI) study is a population‐based prospective cohort study aimed at identifying factors that influence mobility with age located in the Chianti region in Tuscany, Italy (Ferrucci et al., 2000). Briefly, 1,453 individuals were randomly selected based on city registries and ranged in ages from 20 to 102 years old. Overnight fasting blood and plasma samples were stored for genomic DNA extraction and measurement of plasma proteins. The study protocol was approved by the Italian National Institute of Research and Care of Aging IRB and the Medstar Research Institute IRB

### 2.2 SOMAscan^©^ technology

A custom‐designed aptamer profiling platform was used at SomaLogic Inc. to measure protein levels, as previously described (Davies et al., 2012; Emilsson et al., 2018; Hathout et al., 2015). Serum samples were selected from 227 participants (79 centenarians, 83 offspring, and 65 controls) who were alive at least 1 year after the blood draw and were free of major aging‐related diseases at least 1 year from the time of the blood draw (Table 1). The 227 serum samples from the NECS biorepository were assayed with 5,034 SOMAmers. The samples were randomized into analytic batches of 84 samples or less and the plates were assayed as a set, to avoid biases from technical procedures and sample processing. The SOMAscan results passed a quality control assessment for median intra‐ and interassay variability, CV ≤ 15%, similar to variability previously reported in the SOMAscan assays (Candia et al., 2017). Proteomic profiles of 987 plasma samples from InChianti were assayed using the 1.3K SOMAscan Assay at the Trans‐NIH Center for Human Immunology and Autoimmunity, and Inflammation (CHI), National Institute of Allergy and Infectious Disease, National Institutes of Health. The experimental process utilized in the proteomic assessment and normalization was consistent with previously reported experiments with the same technology (Tanaka et al., 2018). The relative abundance of proteins in plasma samples corresponds with the abundance of SOMAmer reagents. The data readout from the SOMAscan‐based proteomics is relative fluorescence units (RFUs) and is directly proportional to the reported relative abundance of SOMAmer reagents.

**Table 1:** Summary of patients' characteristics by *APOE* genotypes

APOE	e2e2	e2e3	e2e4	e3e3	e3e4
N (cent, offs, contr)	7 (0, 5, 2)	44 (14, 19, 11)	4 (1, 0, 3)	143 (56, 48, 39)	24 (4, 10, 10)
Age at blood	70.57 (11.03)	81.73 (17.11)	75 (23.65)	84.72 (18.54)	77.17 (15.26)
Age last contact	78.14 (8.34)	87.95 (14.49)	81.25 (20.11)	90.74 (15.38)	84.75 (13.22)
Year blood	2005.29 (1.7)	2006.75 (3.66)	2006.5 (0.58)	2006.96 (3.81)	2006.33 (2.43)
% Deceased	14 (38)	30 (46)	25 (50)	38 (49)	25 (44)
% Male	29 (49)	41 (50)	25 (50)	38 (49)	29 (46)
Years Education	14.71 (2.29)	15.01 (3.55)	13.5 (2.52)	13.92 (4.34)	15.79 (3.84)
% Dementia	0 (0)	7 (25)	25 (50)	9 (28)	4 (20)
% Angina	0 (0)	7 (27)	0 (0)	11 (31)	0 (0)
% Cancer	14 (38)	14 (35)	2 (0)	32 (47)	25 (44)
% Circulatory	14 (38)	0 (0)	0 (0)	9 (29)	12 (34)
% Congestive	14 (38)	5 (21)	0 (0)	6 (25)	8 (28)
% Diabetes	14 (38)	5 (21)	0 (0)	1 (12)	0 (0)
% MI	0 (0)	7 (25)	0 (0)	6 (25)	8 (28)
% Stroke	14 (38)	11 (32)	0 (0)	13 (33)	17 (38)

Note

Numbers in the first row represent genotype counts, stratify by subject type. Numbers are mean and standard deviation in parenthesis.

Abbreviations: cent, centenarians; MI, myocardial infarction; offs, offspring; contr, controls.

### 2.3 SNP genotyping

*APOE* alleles were inferred from SNPs rs7412 and rs429358 that were either genotyped using real‐time PCR in 2,010 NECS participants, or imputed using IMPUTE2 in participants for whom additional DNA was not available but genome‐wide genotype data were available ([Link]). Genotype data were available for 222 subjects. In the InCHIANTI, *APOE* genotyping of two SNPs rs7412 and rs429358 was completed using TaqMan assay (Applied Biosystems, Inc. [ABI]).

### 2.4 Statistical analysis

We identified three outlier samples in the set of 227 using principal component analysis that were removed from the subsequent analyses. The expression data of the 4,785 proteins were log‐transformed and, for each protein, values in excess of three standard deviations from the mean were removed. The association of each protein with the genotypes of *APOE* were analyzed using a fixed‐effect ANCOVA model, adjusted for sex, age of the serum sample, and age of the participant at blood draw. In particular, the following ANCOVA model was fitted for each of the analytes.

$$\text{log}y_{\text{protein}}∼\beta_{0}+\beta_{\text{gender}}x_{\text{gender}}+\beta_{\text{year}}x_{\text{year}}+\beta_{\text{age}}x_{\text{age}}+\sum_{g}\beta_{g}x_{g},$$

where the dummy variables *x*_g_ denote carriers of one of the *APOE* genotype $g=e_{2}e_{2},e_{2}e_{3},e_{2}e_{4},e_{3}e_{4}$, and *β*_g_ represents the log‐transformed fold change of the analyte comparing carriers of the genotype *g* relative to carriers of the common genotype *e*_3_ *e*_3_.

We selected significant proteins based on the *F* test, with 4 and 214 degrees of freedom, and used a false discovery rate (FDR) < 0.01 as level of significance to correct for multiple testing with Benjamini–Hochberg. Qvalues were calculated using the qvalue package in R. For comparison with published protein quantitative trait loci (pQTL), we analyzed the association between the two SNPs rs7412 and rs429358 and the expression of the significant proteins in the 222 NECS samples using regression of the log‐transformed expression adjusted for sex and age at blood draw. We analyzed the associations of SNPs rs6857, rs769449, and rs2075650 in the same locus of *APOE* and the level of the significant proteins, after adjusting for the *APOE* genotype, sex, and age at blood draw. The rationale for choosing these three SNPs was that they have been reported in the longevity literature as possible genetic variants of longevity and healthy aging, with effects independent of the *APOE* alleles (Sebastiani et al., 2018). We correlated the expression of the log‐transformed proteins associated with APOE genotypes with longitudinal change of TICS using linear regression adjusted for *APOE* genotypes, sex, age, and education, and we estimated the regression coefficients using the generalized estimating equations to account for repeated measures.

### 2.5 Replication

The results were replicated in three independent datasets.

#### 2.5.1 Replication in InCHIANTI

Five of the 16 proteins were measured in 987 plasma samples from participants of InCHIANTI using the same SOMAscan technology and a platform with 1,301 proteins. The RFU values were natural log‐transformed, and outliers outside 3SD were removed. The association between the log‐transformed level of the five proteins and APOE genotypes were analyzed using regression adjusted for age, sex and study site (Chianti or Ripoli).

#### 2.5.2 Replication in published pQTLs in plasma and serum

We extracted all significant associations between SNPs rs7412 and rs429358 and cis‐ and trans‐proteins discovered in 3,301 plasma samples described in (Sun et al., 2018), and in 5,457 serum samples described in (Emilsson et al., 2018). We compared the results with the association between the two SNPs and the expression of the 16 proteins in the 222 NECS samples. The results of the comparison are in Table 4.

### 2.6 In silico validation

We evaluated the 16 genes corresponding to the *APOE* signature in gene expression data of postmortem brain tissue from three brain regions (PC, prefrontal cortex; VC, visual cortex; and CB, cerebellum) from 129 LOAD patients and 101 healthy controls (Zhang et al., 2013), for a total of 690 profiles. We used the geneset variation analysis method (GSVA) implemented in the R package gsva (Hanzelmann, Castelo, & Guinney, 2013) to produce a summary APOE signature score per subject, which can be interpreted as the level of expression of the gene signature. GSVA is a test of geneset enrichment that takes as input our gene‐by‐sample expression data matrix (we mapped each protein to a gene symbol) and generates a geneset‐by‐sample “enrichment score” matrix, where the (i,j) entry denotes the modified Kolmogorov–Smirnov (KS) test statistic that measures the enrichment of the i^th^ geneset's genes in the j^th^ sample. The enrichment scores are in the range [−1.0;+1.0], with a positive (negative) score indicating coordinated up‐ (down‐)regulation in the sample. In our analysis, we used two genesets, one determined by the nine proteins that increase levels in carriers of the *e*_2_ allele (UP), and one determined by the seven proteins that increase levels in carriers of the *e*_4_ allele and decrease in carriers of the *e*_2_ allele (DN). The final APOE enrichment score was computed as the difference of the UP and DN scores (UP – DN), thus taking values in the range [−2;+2]. The score distributions of LOAD patients and controls were compared using Wilcox test and ANOVA. The comparisons were performed using samples *across* all brain tissues, as well as *within* specific brain region tissues. Additionally, the skewedness of the LOAD patients' distribution—that is, the over‐representation of LOAD patients among lower APOE scores—was assessed by a nonparametric one‐sample KS test of deviation from the uniform distribution. The KS score and its significance were computed with the R function ks.test.

All analyses were conducted in R V3.5.

## 3 RESULTS

Table 1 displays characteristics of the NECS patients included in the analysis. We enriched the sample selection of carriers of the *e*_2_ *e*_2_ genotype of *APOE* that is more prevalent in healthy agers and centenarians (Sebastiani et al., 2018). The ages of study participants varied between 45 years and 114 years, but mean age per genotype groups was comparable. By design, the participants included in this study were healthy and survived at least 1 year beyond the blood draw.

### 3.1 Discovery

**Figure 1:**
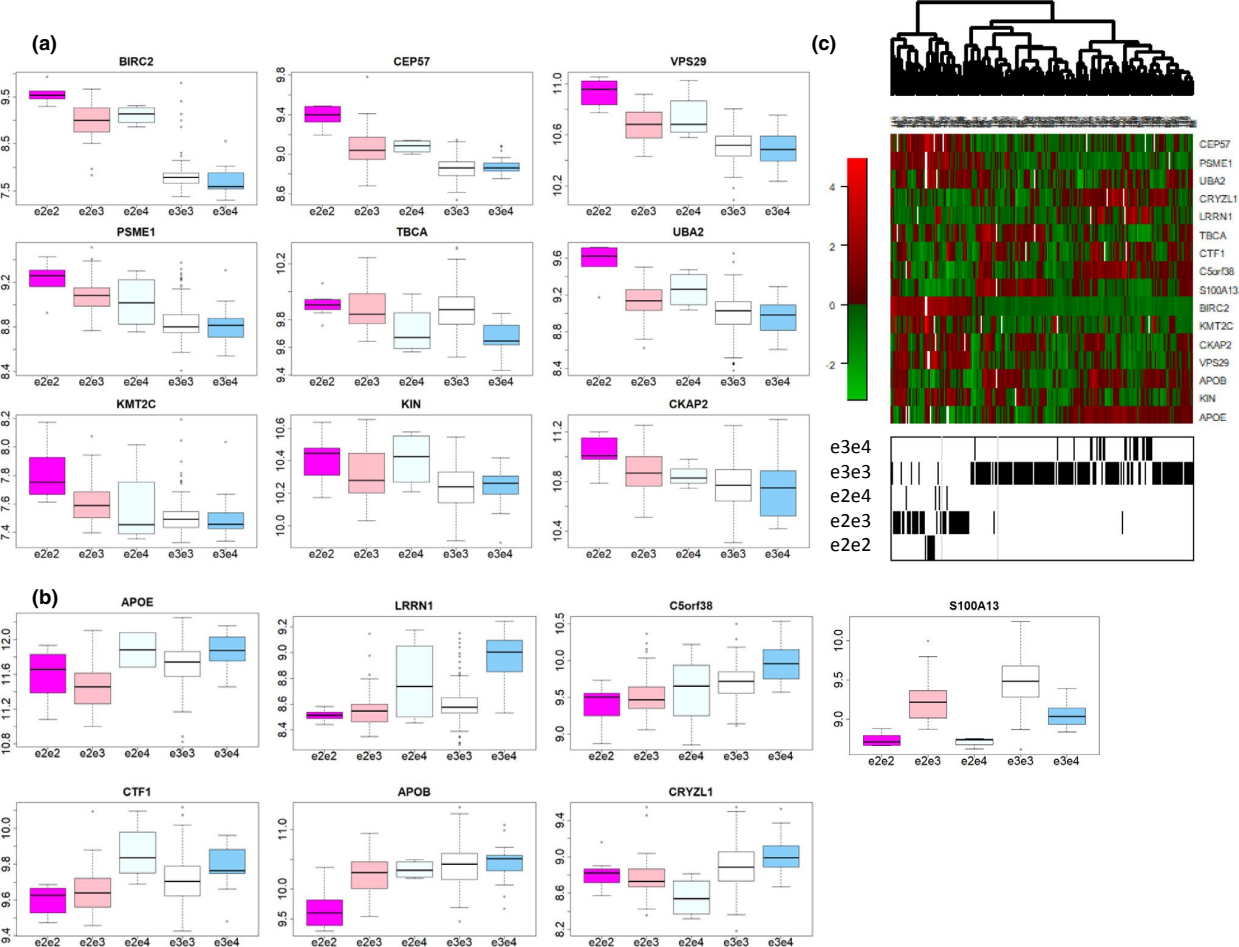
Distribution of protein intensity in log‐scale by *APOE* genotypes. a) Boxplot of nine proteins that increase expression in carriers of the *e*_2_. b) Boxplot of seven proteins that increase expression in carriers of the *e*_4_. c) Heatmap of the *APOE* signature. Rows are standardized proteins in the signature, columns are samples and the barcode represents the *APOE* genotypes sorted after hierarchical clustering of the samples

Table 2 shows the list of 16 proteins significantly associated with the *APOE* genotypes at 1% FDR. Table S1 shows the results of the analysis for all 4,785 analytes. The 16 proteins that passed the significance threshold include 9 overexpressed in carriers of the *e*_2_ allele (Figure 1a), and 7 overexpressed in carriers of the *e*_4_ allele (Figure 1b). Besides APOE and APOB, the other proteins have not previously been reported as directly associated with the *APOE* genotypes. The pattern of APOB expression by *APOE* genotype is consistent with results published in (Muenchhoff et al., 2017) and (Soares, Potter, & Pickering, 2012), and the rare *e*_2_ *e*_2_ genotype was associated with the lowest APOB level. The level of the APOE protein probe included in this list is lowest in carriers of *e*_2_ and increases in carriers of *e*_3_ and *e*_4_. Interestingly, the effect of the *e*_2_ allele on most proteins was additive in the log‐scale, as shown by the almost linear change of log‐expression for ordered genotypes in Figure 1a,b. The genetic effect was recessive on APOB and dominant on BIRC2.

**Table 2:** Signature of 16 biomarkers associated with APOE genotypes

SomaID	Uniprot	geneID	e2e2	e2e3	e2e4	e3e4	*p*	q
10046‐55	Q13490*	BIRC2	5.87	3.23	3.67	0.90	1.55E−61	7.42E−58
11276‐1	Q86XR8*	CEP57	1.62	1.23	1.22	1.01	1.96E−28	4.68E−25
7223‐60	Q99584	S100A13	0.51	0.78	0.47	0.67	2.69E−23	4.30E−20
11293‐14	Q6UXK5	LRRN1	0.89	0.96	1.17	1.40	1.17E−19	1.40E−16
14318‐1	Q9UBQ0	VPS29	1.55	1.18	1.26	0.98	2.84E−19	2.72E−16
5918‐5	Q06323*	PSME1	1.51	1.27	1.22	0.99	5.23E−19	4.17E−16
2418‐55	P02649*	APOE	0.86	0.77	1.15	1.16	9.19E−11	6.28E−08
12501‐10	O75347*	TBCA	1.08	1.02	0.88	0.83	1.60E−10	9.57E−08
12500‐88	Q9UBT2*	UBA2	1.77	1.13	1.31	0.98	5.44E−10	2.89E−07
6378‐2	Q86SI9	C5orf38	0.73	0.84	0.88	1.29	7.68E−10	3.67E−07
13732‐79	Q16619	CTF1	0.94	0.95	1.20	1.11	9.63E−09	4.19E−06
11402‐17	Q8NEZ4*	KMT2C	1.33	1.11	1.06	0.99	2.15E−08	8.58E−06
14643‐27	O60870*	KIN	1.23	1.08	1.22	1.02	3.10E−07	0.000114
2797‐56	P04114*	APOB	0.50	0.86	0.97	1.07	3.36E−06	0.001148
9207‐60	O95825	CRYZL1	0.89	0.88	0.70	1.11	7.07E−06	0.002257
5345‐51	Q8WWK9	CKAP2	1.33	1.12	1.08	0.96	8.12E−06	0.002429

Note

Columns e2e2, e2e3, e2e4, e3e4 report fold change of protein level relative to e3e3. *P* is *p*‐value from *F* test with 4 and 214 degrees of freedom, after adjusting for sex, age at blood draw, and length of sample storage. Q are qvalues ccorresponding to 1% FDR.

* Proteins that include at least one coiled coil domain.

The heatmap in Figure 1c shows good separation of samples by *APOE* genotypes and suggests that the 16 proteins together cluster in patterns that correlate with the *APOE* genotypes. The 16 proteins have a variety of functions including regulation of cell proliferation, cell surface receptor, protein binding, and immune system, and a complete description of their functions extracted from the human protein atlas (https://www.proteinatlas.org/) is in Table S2. We annotated the list of 16 proteins in the signature using DAVID (Huang et al., 2009), with background restricted to the list of proteins assayed in the SOMAscan array. The analysis showed that the signature was enriched with proteins that contain at least one coiled coil domain (FDR < 0.1%) that are highlighted in Table 2.

### 3.2 Replication

We investigated the association between the *APOE* genotypes and levels of expression of five of the 16 proteins that were also measured in 987 plasma samples of InCHIANTI participants. The results in Table 3 show significant replication of the associations with APOE, APOB, and CRYZL1, with consistent effects, while PSME1 and CKAP2 did not show any variation by APOE genotypes in this set.

**Table 3:** Replication in inCHIANTI (Plasma)

		NECS (Serum)					inCHIANTI (Plasma)			
SomaID	geneID	e2e2	e2e3	e2e4	e3e4	*p* ^+^	E2	*p*	E4	*p* ^++^
**5918‐5**	PSME1	1.51	1.27	1.22	0.99	5.23E−19	1.03	.507676	1.02	.574456
**2418‐55**	**APOE**	0.86	0.77	1.15	1.16	9.19E−11	0.69	9.62E−17	1.25	6.81E−08
**2797‐56**	**APOB**	0.5	0.86	0.97	1.07	3.36E−06	0.78	7.35E−11	1.12	.000889
**9207‐60**	**CRYZL1**	0.89	0.88	0.7	1.11	7.07E−06	0.79	3.00E−11	1.11	.001088
**5345‐51**	CKAP2	1.33	1.12	1.08	0.96	8.12E−06	1.00	0.991364	1.00	.987645

Note

Columns e2e2, e2e3, e2e4, e3e4 report fold change of protein level relative to e3e3, and *p* ^+^ is *p*‐value from *F* test with 4 and 214 degrees of freedom after adjusting for sex, age at blood draw, and length of sample storage. Columns E2 and E4 report fold changes for genotype groups E2 = e2e2 or e2e3, E4 = e2e4, e3e4, e4e4, relative to e3e3 and *p* and *p* ^++^ are *p*‐values from *T* test for age, sex and study site.

Recent protein scans of human serum and plasma conducted with SOMAscan arrays in 3,301 plasma samples (Sun et al., 2018), and in 5,457 serum samples (Emilsson et al., 2018) published cis‐ and trans‐protein associations of the two SNPs rs7412 and rs429358 that define the APOE alleles $\left(e_{2}:\text{rs}7412=T,\text{rs}429358=\mathrm{T;}e_{3}:\text{rs}7412=C,\text{rs}429358=T;e_{4}:\text{rs}7412=C,rs429358=C\right)$. We estimated the associations between the two SNPs and the 16 proteins listed in Table 2 in the 222 NECS serum samples, and Table 4 compares the results with the results published in serum (Supplement Heppner et al., 2015 in (Emilsson et al., 2018)), and plasma (Supplement table in (Sun et al., 2018)). For all but APOB, we found a significant association with rs7412 or rs429358 in the NECS serum samples, and all the associations were significantly replicated in either one or both studies with consistent effects. Note that the protein scan in plasma used a reduced SOMAscan array covering less than 3,000 proteins, thus limiting the replication set, while the protein scan in serum used a SOMAscan array comparable to the one used in our study. The genetic effects reported in (Emilsson et al., 2018) were estimated after using a Yeo–Johnson transformation of the protein data to improve normality; hence, the effects are not directly comparable to our analysis in which we used a log‐transformation of the protein data. However, the directions of effects are all consistent. Interestingly, this analysis showed a significant replication of the effect of the T allele of rs7412 with levels of PSME1 and CKAP2 that is consistent with overexpression of these two proteins in carriers of the $e_{2}$ allele but failed to replicate in InCHIANTI.

**Table 4:** SNP‐protein associations and their replication in two independent sets

				NECS			Science (Serum)		Nature (Plasma)		
	SomaID	Uniprot	geneID	beta	SE	*p*	beta	*p*	beta	SE	*p*
rs7412/ T	10046‐55	Q13490	BIRC2	1.21	0.07	2.41E−38	1.70	1.39E−302			
	11276‐1	Q86XR8	CEP57	0.21	0.02	4.07E−15	0.90	7.42E−70			
	5918‐5	Q06323	PSME1	0.26	0.03	1.14E−13	0.66	1.63E−41	1.06	0.04	4.30E−137
	14318‐1	Q9UBQ0	VPS29	0.17	0.03	2.10E−09	1.25	1.51E−147	1.69	0.04	0
	2418‐55	P02649	APOE	−0.23	0.05	2.67E−06	−0.80	2.73E−56			
	11402‐17	Q8NEZ4	KMT2C	0.11	0.03	5.19E−05	0.38	1.23E−13	.59	0.05	6.90E−39
	9207‐60	O95825	CRYZL1	−0.17	0.04	8.70E−05	−0.71	9.61E−47			
	14643‐27	O60870	KIN	0.09	0.02	.00014	0.50	1.67E−23			
	7223‐60	Q99584	S100A13	−0.19	0.05	.00017	−0.83	1.85E−62			
	6378‐2	Q86SI9	C5orf38	−0.18	0.05	.00109	−1.02	1.64E−92			
	5345‐51	Q8WWK9	CKAP2	0.12	0.04	.00171	0.43	7.53E−18			
	12500‐88	Q9UBT2	UBA2	0.10	0.04	.01052	1.47	3.74E−210			
	2797‐56	P04114	APOB	−0.12	0.07	.06623	−0.57	9.23E−30			
rs429358/T	11293‐14	Q6UXK5	LRRN1	−0.26	0.04	8.01E−10			−1.18	0.03	0
	13732‐79	Q16619	CTF1	−0.15	0.02	5.56E−09			−.80	0.03	3.50E−148
	12501‐10	O75347	TBCA	0.16	0.03	2.54E−08			1.34	0.02	0

Note

NECS: beta coefficients and standard errors estimated from linear regression of log‐transformed protein data, adjusted for age and sex.

Science (Serum): beta coefficients estimated from linear regression of Yeo–Johnson transformation of protein data (Emilsson et al., 2018). Cis‐ and trans‐effects available only for rs7412.

Nature (Plasma): beta coefficients and standard errors estimated from fixed‐effect inverse‐variance meta‐analysis of two cohorts (Sun et al., 2018). Cohorts specific results reported beta coefficients of SNPs on log‐transformed protein levels, adjusted for sex, age, BMI, and additional covariates.

Data of missing proteins were not available.

To evaluate whether additional SNPs in the *APOE* locus could explain the association between the *APOE* genotypes and the 16 proteins in the signature, we analyzed the association between the 16 proteins and each of the SNPs rs6857, rs769449, and rs2075650, adjusting for sex, age at blood draw, and the *APOE* genotypes. While the association between the *APOE* genotypes and each of the 16 proteins remained significantly associated, none of the SNPs rs6857, rs769449, and rs2075650 was a significant pQTL for these proteins in the multi‐SNP analysis (Table S3).

### 3.3 Association of the APOE signature to LOAD status in brain tissues

**Figure 2:**
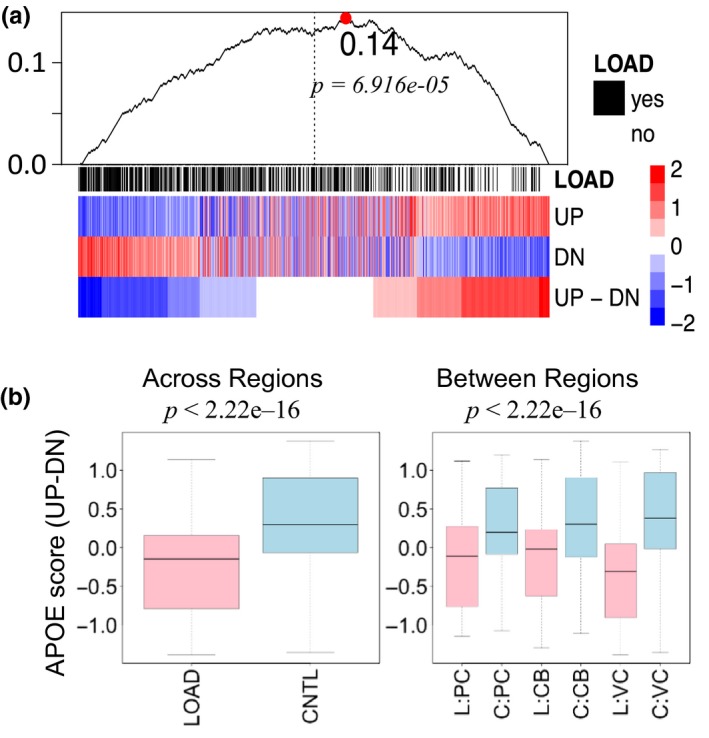
Projection of the APOE signature in brain RNA samples. a) Heatmap of the signature score generated with GSVA in 690 brain samples. UP‐DN = all 16 genes; UP: nine genes with proteins that increase levels in carriers of the *e*_2_ allele; DN: seven genes with proteins that increase levels in carriers of the *e*_4_ allele. Displayed above the heatmap is the plot corresponding to the KS test assessing the significance of the skewedness in the distribution of LOAD patients toward low levels of the UP‐DN score (see Methods). b) Boxplots of the signature score in brains of the LOAD patients (pink) and healthy controls (blue). Left: all brain samples together; Right: samples stratified by brain regions. *p* Values from *F* test from ANOVA

We evaluated the *APOE* signature in gene expression data of postmortem brain tissue from 129 LOAD patients and 101 healthy controls (Zhang et al., 2013) to test the hypothesis that the serum protein signature corresponds to distinct gene expression signatures in brains of LOAD patients and healthy controls. Figure 2a shows the distribution of the gene expression signature scores generated with R‐GSVA using all 16 genes in the 690 brain samples (UP – DN), and the two sets of genes corresponding to the nine proteins with increased levels in carriers of the *e*_2_ allele (UP) and the seven proteins with increased levels in carriers of the *e*_4_ allele and decrease in carriers of the *e*_2_ allele (DN). The heatmap shows clear separation between gene expression signatures of LOAD and healthy patients, with the LOAD subjects significantly skewed toward the low values of the signature score (black ticks above heatmap, Kolmogorov–Smirnov *p*‐value < .00007). The boxplots of the distribution of the signature score in brains of LOAD patients (pink) and healthy controls (blue) in Figure 2b confirm that the LOAD subjects have a significantly lower signature score than the controls, both across and within brain regions (*p* < 2.2E−6).

### 3.4 Effect on cognitive function

**Figure 3:**
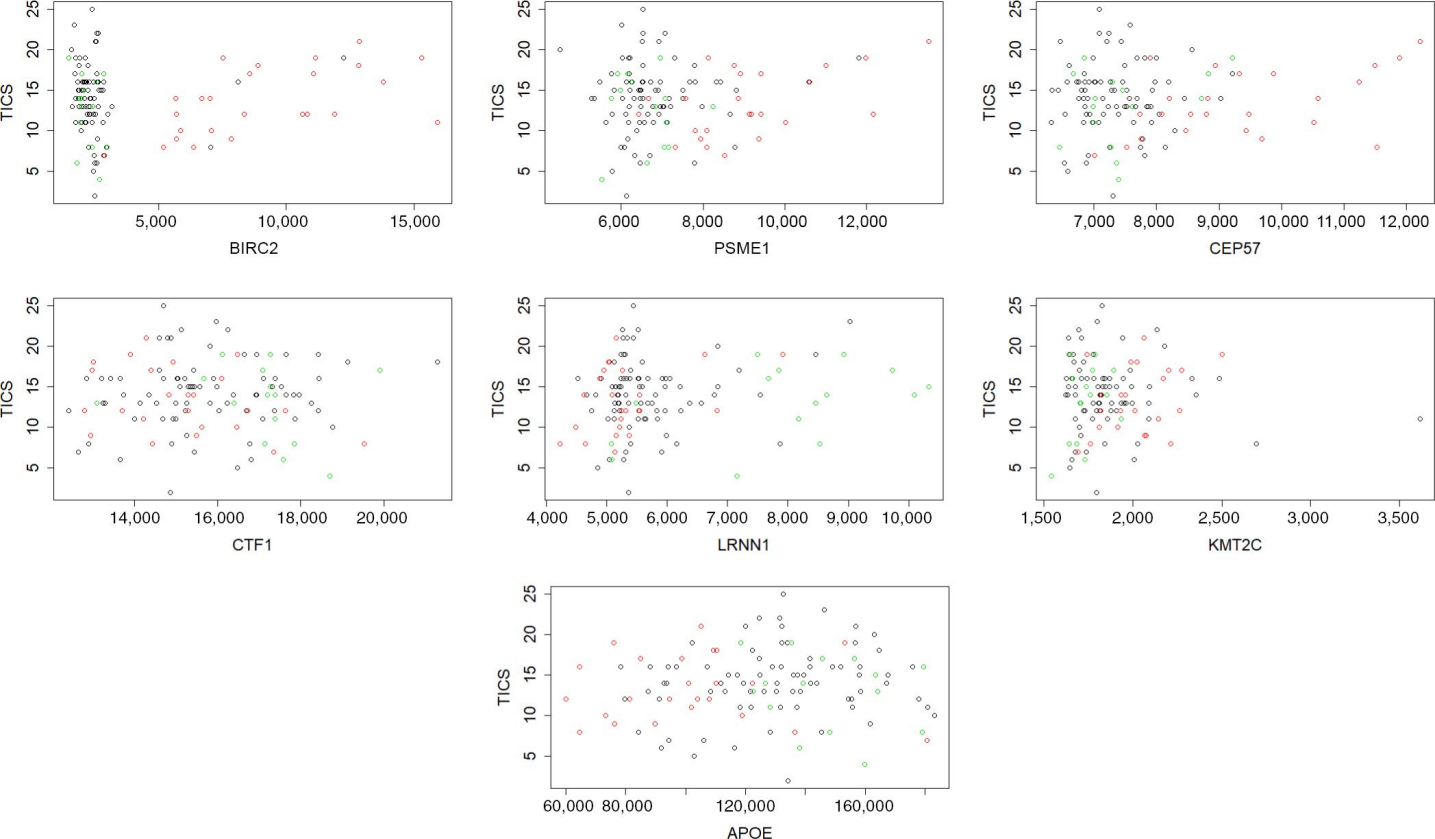
Scatter plots of TICS score (y‐axis) versus RFU of the seven proteins listed in Table 5. Red: carriers of E2, green: carriers of E4, black: carriers of E3. APOE genotype groups were defined as in 5

We examined the association of the 16 proteins with level and rate of change of cognitive function in longitudinally administered TICS in the 148 centenarian offspring and controls included in the study. For each individual, we had up to five repeated assessments of TICS. In preliminary analyses, we modeled the effect of the 16 individual proteins on the longitudinal trajectories of TICS using linear regression. None of the proteins or the *APOE* genotypes were significantly associated with the TICS score after adjusting for age and sex. However, when we conducted analyses in strata of the data defined by *APOE* genotype groups, six proteins showed nominal significant association with TICS score (Table 5, *p* < .05), and PSME1 showed borderline significant association with TICS score (*p* = .0751). The associations between BIRC2, CEP57, KMT2C, and APOE remained significant even after correction for multiple testing (*p* < .05/16 = 0.003). Increasing levels of BIRC2, PSME1, CEP57, and LRRN1 were associated with increasing TICS score in carriers of one or more $e_{2}$ alleles, while increasing values of CTF1 were associated with decreasing TICS score in the same genetic background (Figure 3). The analysis also showed that in carriers of one or more $e_{4}$ alleles increasing values of KMT2C, CEP57, and LRRN1 were associated with increasing TICS score, while increasing levels of APOE were associated with decreasing TICS score. We also fitted models with interactions between protein levels and age at TICS to test the hypothesis that levels of proteins in the signature modify the rate of change of TICS score, but none of the interactions reached statistical significance. The interaction between BIRC2 levels and age reached borderline statistical significance (*p* = .057) in carriers of one or more *e*_2_ alleles.

**Table 5:** Association of proteins in the APOE signature with cognitive function

	E2			E4		
	beta	SE	*p*	beta	SE	*p*
BIRC2^a^	4.02	1.27	.0016	−2.67	2.38	.2617
PSME1^b^	5.99	3.36	.0751	2.97	5.48	.5875
CEP57^c^	8.55	4.40	.0519	11.74	3.47	.0007
CTF1^c^	−12.80	6.27	.0414	−3.34	5.62	.5526
LRRN1^c^	10.59	4.09	.0096	3.69	1.94	.0574
KMT2C^c^	5.76	7.72	.4561	21.12	4.51	.0000
APOE^c^	0.41	3.07	.8929	−9.54	2.49	.0001

Note

Columns 2—4: association conditionally on APOE = E2, Columns 5—7: associations conditionally on APOE = E4. Beta, SE and *p* represent the effect, robust standard error and *p*‐value of the log‐transformed protein on reduced Telephone Interview for Cognitive Status (TICS), using generalized estimating equations.

a E2 = $e_{2}e_{2},e_{2}e_{3},e_{2}e_{4}$; E3 = $e_{3}e_{3}$; E4 = $e_{3}e_{4}$.

b E2 = $e_{2}e_{2},e_{2}e_{3}$; E3 = $e_{3}e_{3}$; E4 = $e_{3}e_{4}$.

c E2 = $e_{2}e_{2},e_{2}e_{3}$; E3 = $e_{3}e_{3}$; E4 = $e_{2}e_{4},e_{3}e_{4}$.

## 4 DISCUSSION

Comprehensive measurement of the proteome in a large number of samples has been challenging. The SomaLogic aptamer‐based technology has emerged in the last few years as a robust, high throughput assay for quick and scalable measurement of protein levels (Davies et al., 2012). Recent publications have shown the richness of the proteome of human plasma and serum and the fact that proteins expressed in serum can be helpful to detect potential regulatory mechanisms (Emilsson et al., 2018), as well as to discover accessible diagnostic and prognostic biomarkers (Hathout, 2015). In this work, we combined access to the largest plex aptamer‐based proteomic platform available, and a relatively large number of serum samples from a unique population enriched for carriers of *e*_2,_ healthy agers and extreme survivors to discover a novel protein signature of the *APOE* genotypes. The signature includes known cis‐ and trans‐proteins associated with *APOE* genotypes (Muenchhoff et al., 2017), namely APOB and APOE, and 14 proteins not previously associated with specific *APOE* genotypes. We replicated part of the results in plasma proteins that were profiled using a smaller platform based on the same technology (Tanaka et al., 2018), and we also showed agreement between our results and pQTLs in serum and plasma discovered in the *APOE* locus in much larger studies (Emilsson et al., 2018; Sun et al., 2018).

Some of the proteins picked up are particularly noteworthy. The signature includes APOE and APOB that are specifically expressed in brain, liver, gastrointestinal tissues, and skin, while the other 14 proteins are expressed in multiple tissues and have a variety of biological functions described in Table S2. Baculoviral IAP repeat containing two (BIRC2, also known as cIAP1) is a member of a family of proteins that inhibit apoptosis (Marivin, Berthelet, Plenchette, & Dubrez, 2012; Varfolomeev et al., 2007) and was shown to be target for neuroprotection in rat brains after stroke (Huang et al., 2015). BIRC2 is a regulator of the noncanonical NF‐kappaB pathway (Mak et al., 2014), though a positive regulator of canonical NF‐kappaB signaling (Hinz et al., 2010). Alzheimer's disease has been associated with inflammation in the brain (Heppner, Ransohoff, & Becher, 2015; Kinney et al., 2018), so the finding that this ligase is associated with better outcomes is of interest.

S100 calcium binding protein A13 (S100A13) is a member of the S100 family of proteins involved in several biological functions and interacts with the receptor for advanced glycation end products (RAGE) (Rani, Sepuru, & Yu, 2014). RAGE activation is associated with neuroinflammation and neurodegeneration, and although the mechanism remains unclear, there is strong evidence supporting a role of RAGE in several neurodegenerative diseases including AD (Ray, Juranek, & Rai, 2016). VPS29, retromer complex component, belongs to a group of vacuolar protein sorting (VPS) genes that may be related to AD pathology (Vieira et al., 2010). Tubulin folding cofactor A (TBCA) is involved in the pathway leading to correct folding of beta tubulin. Some literature on AD pathogenesis suggests an interaction between tubulin and tau (Puig, Ferrer, Luduena, & Avila, 2005; Salama et al., 2018) and the association between TBCA and *APOE* genotypes suggests a mechanism of genetic regulation. CRYZL1 is the product of the gene *CRYZL1* in chromosome 21 and includes a NAD(P)H binding site. Interestingly, chromosome 21 trisomy is associated with higher risk for early onset of AD, and NAD(P)H oxidase is upregulated in AD brain (Block, 2008). LRNN1 (leucine‐rich neuronal protein) is a secreted protein that has been previously associated with Alzheimer's disease by RNA (Bai et al., 2014). In chicks, LRRN1 is required for the formation of the midbrain (Tossell et al., 2011), but it seems its function is to define neuronal boundaries, so perhaps its function is inhibitory for differentiated neurons. LRNN1 is also highly expressed in unfavorable neuroblastoma, which also suggests a negative role in neuronal differentiation, though a positive inducer of proliferation (Hossain et al., 2012). Another secreted protein that appears to be upregulated in carriers of the *e*_4_ allele is cardiotrophin‐1 (CTF1). This result is surprising since two separate papers found overexpression of this gene to be protective in mice models of Alzheimer's (Wang et al., 2013; Wang, Liu, Liu, Li, & Wang, 2017). Our findings do not suggest this, although of course the upregulation might be "compensatory." Cardiotrophin‐1 acts through the IL‐6 receptor and is therefore an activator of inflammatory signaling. Perhaps it is not surprising after all to learn that a pro‐inflammatory molecule is positively associated with Alzheimer's disease.

Nine of the 16 proteins in the signature include a coiled coil domain. Compared to 472 proteins with a coiled coil domain in the list of annotated 4,127, the inclusion of nine in 16 represents an almost fivefold enrichment (*p*‐value .006 from Fisher's exact test). Coiled coil domains are potentially involved in aggregation of amyloid (Fiumara, Fioriti, Kandel, & Hendrickson, 2010), and this suggests that a possible neuroprotective mechanism associated with the *e*_2_ could be to limit accumulation of β‐amyloid. Another surprising aspect of the result is that some of these proteins are thought to be intracellular. It is not uncommon, however, to find cytoplasmic proteins in sera by SOMAscan (Geyer et al., 2016) For example, BIRC2 encodes an E3 ubiquitin ligase, which does not contain a signal sequence but its upregulation in the blood may be a general indicator of increased expression body‐wide.

Emilsson and coauthors used a SOMAscan platform with 4,785 proteins to profile the serum of 5,457 serum samples and used scale‐free network analysis to show that serum protein clusters in a small number of modules (Emilsson et al., 2018). They showed that a cluster of SNPs in the *APOE* locus is associated with a lipoprotein enriched module of 27 serum proteins that share APOE, TBCA, APOB, S100A13, CRYZL1, C5orf38 with our signature (Emilsson et al., 2018). Our analysis shows that the overlapping associations are attributable to the specific *APOE* genotypes, rather than other genetic variants in the same locus. In addition, Emilsson's work suggests that protein modules in serum have a 37.3% agreement with gene expression signatures in various tissues. Consistent with their results, our analysis shows that the expression of the genes associated with the proteins in the *APOE* signature produce brain transcriptional profiles that distinguish AD patients from healthy controls. This result suggests that the protein signature in serum could be a candidate biomarker for AD resistance, diagnosis and possibly prognosis. However, the value of the signature as serum‐biomarker of AD needs to be assessed and replicated in larger samples to establish its clinical value.

The small sample size of this study and the selection of healthy subjects gave limited power to correlate the protein signature with aging markers. However, we are able to show that seven of the proteins in the *APOE* signature are associated with TICS score in particular genetic backgrounds, suggesting that these proteins have a predictive value in addition to the putative neuroprotective role of the $e_{2}$ alleles of APOE and could be novel targets for neuroprotective interventions. For example, the pattern of expression of BIRC2 by *APOE* genotypes (Figure 1) and the strong association between BIRC2 and TICS score in carriers of one or more *e*_2_ alleles (Table 5) suggest that this protein may be expressed only in carriers of one or more *e*_2_ alleles and that additional factors contribute to its varying expression level that positively correlates with better cognitive function. The patterns of PSME1 and CEP57 and the positive correlation with TICS in carriers of one or more *e*_2_ alleles suggest that compounds that increase these protein levels could also lead to neuroprotection. The pattern of association between LRRN1 and TICS score however is less clear since in both carriers of the *e*_2_ alleles and the *e*_2_ alleles, increasing values of LRRN1 predict higher TICS scores. This protein needs more in‐depth characterization in order to understand its role relative to *APOE* genotypes and cognitive status.

The advantage of working with serum and plasma proteins is that blood is easily accessible and therefore ideal for biomarker discovery, but the lack of tissue specificity may challenge the understanding of the biological mechanisms. Recently established bioinformatic resources of human proteins, for example, the human protein atlas ([Link]; Thul & Lindskog, 2018; [Link]), provide detailed annotation of protein expression in more than 80 tissues and cell types and can be used to help generate hypotheses of the biological mechanisms that translate genetic variants into expressed phenotypes. A constant debate in the field is whether serum (lacks clotting factors) or plasma should be used. We could replicate 9 of the associations discovered between serum proteins and *APOE* genotypes in plasma, but no data were available to test the association of BIRC2, CEP57, KIN, S100A13, C5orf38, CKAP, and UBA2 in plasma. Soares et al (Soares et al., 2012) identified a signature of *APOE* genotypes in plasma that included APOE and APOB, and additional proteins CXCL9 and IL13. Levels of IL13 were not associated with *APOE* genotypes in any of the SOMAmers included in the platform. We detected a statistically significant association between levels of CXCL9 and *APOE* genotypes (*p* = .0015) but with inconsistent effects.

There is a vast literature about circulating biomarkers of AD and *APOE* genotypes in cerebral spinal fluid (CSF) and blood (Huynh & Mohan, 2017; Soares et al., 2012), but past analyses have been limited by panels of small numbers of biomarkers. Our work explores for the first time a large spectrum of serum proteins in a relatively large sample of *APOE e*_2_ allele carriers. The results show that several circulating proteins are in cis and trans with *APOE* genotype, and the reproducibility of the results in multiple independent studies provides strong evidence that the results are real, although more work is needed to show that the results are biologically meaningful and clinically useful. Specifically, three important issues remain to be addressed: (a) independent validation of the SOMAscan results using an alternative proteomic technology is necessary to validated the proteins in the signature; (b) these initial results will need to be followed up experimentally, to understand whether the proteins that are differentially regulated in carriers of the $e_{2}$ allele can point to neuroprotective treatments that could delay/avoid cognitive decline; and (c) whether the *APOE* protein signature can be used for noninvasive diagnostic and prognostic biomarkers of cognitive decline or protection. The annotation available for some of the proteins in the signature suggests that more in‐depth proteomics is needed to better characterize the post‐translational modifications that could modify their molecular functions. In addition, the assessment of the potential clinical value of the signature will require the development of assays to measure the signature on large number of samples.

## CONFLICT OF INTEREST

M.M., L.L.J., and D.J.G are employees and stockholders of Novartis.
